# Type 1 diabetes mellitus in the context of high levels of rural deprivation: differences in demographic and anthropometric characteristics between urban and rural cases in NW Ethiopia

**DOI:** 10.3389/fcdhc.2023.1298270

**Published:** 2024-01-29

**Authors:** Shitaye A. Balcha, David I. Phillips, Elisabeth R. Trimble

**Affiliations:** ^1^ Department of Internal Medicine, Gondar University Hospital, Gondar, Ethiopia; ^2^ Medical Research Council (MRC) Lifecourse Epidemiology Unit, University of Southampton, Southampton General Hospital, Southampton, United Kingdom; ^3^ Centre for Public Health, Institute of Clinical Science, Queen’s University Belfast, Belfast, United Kingdom

**Keywords:** type 1 diabetes, urban rural, anthropometric and demographic differences, resource poor communities, Ethiopia

## Abstract

**Background:**

While there is increasing evidence for an altered clinical phenotype of Type 1 diabetes in several low-and middle-income countries, little is known about urban-rural differences and how the greater poverty of rural environments may alter the pattern of disease.

**Objective:**

Investigation of urban-rural differences in demographic and anthropometric characteristics of type 1 diabetes in a resource-poor setting.

**Research design and methods:**

Analysis of a unique case register, comprising all patients (rural and urban) presenting with Type 1 diabetes over a 20 yr. period in a poor, geographically defined area in northwest Ethiopia. The records included age, sex, place of residence, together with height and weight at the clinical onset.

**Results:**

A total of 1682 new cases of Type 1 diabetes were registered with a mean age of onset of 31.2 (SD 13.4) yr. The patients were thin with 1/3 presenting with a body mass index (BMI) <17kg/m^2^. There was a striking male predominance of cases when clinical onset was between 20 and 35 yr., this was more marked in the very poor rural dwellers compared to the urban population. While most patients with Type 1 diabetes presented with low BMIs and reduced height, stunting preferentially affected rural men.

**Conclusions:**

These data have led to the hypothesis that complex interactions among poor socioeconomic conditions in early life affect both pancreatic function and the development of autoimmunity and provide a possible explanation of the unusual phenotype of Type 1 diabetes in this very poor community.

## Introduction

There is increasing evidence that the clinical presentation of insulin dependent, type 1 diabetes in several low- and middle-income countries (LMIC) is different from the ‘classical’ presentation in affluent countries; this is particularly the case in sub-Saharan Africa (SSA). Despite the enormous genetic diversity and wide range of socio-economic conditions, a number of consistent features have been described. First, with respect to age of onset, studies in Ethiopia (1) and other widely separated areas of SSA (2, 3) have reported a lower proportion presenting in childhood compared with industrialized countries. Second, diabetes-related autoantibody profiles are different in SSA, even when measured shortly after the clinical onset of the disease (3, 4). Third, in extremely poor socioeconomic conditions a male excess among presenting cases has been reported (1, 5, 6).

These phenotypic differences are somewhat heterogeneous (7), poorly understood, and may depend on the degree of deprivation. Consequently, they have led to the suggestion that the poor socioeconomic conditions and related undernutrition prevailing in these communities may play a role in the pathogenesis of the disease (6, 8, 9). Despite there being a longstanding body of evidence from animal studies that undernutrition can be a cause of diabetes through a reduction in total β-cell mass (10) and that the combination of early-life undernutrition and later development of obesity is implicated in the development of type 2 diabetes in humans (11), there is sparse evidence that poor nutrition influences the development of type 1 diabetes. However, in the context of resource-poor countries such as Ethiopia where rural undernutrition typically occurs throughout the life course there was some evidence for a nutritional effect from an early study showing stunting and skeletal disproportion in male cases at the clinical onset of type 1 diabetes when compared with non-diabetic controls from similar socioeconomic backgrounds (8).

In Ethiopia as in other countries in SSA there are marked differences between urban and rural populations. People living in rural areas are often poor, tend to have a more active lifestyle and consume more restricted traditional diets while urban populations have differences in lifestyle, diet and socioeconomic conditions. The macro and micronutrient deficiencies which give rise to stunting (12, 13) are often worse in rural areas of LMIC (14). Additionally, and for reasons not completely understood, there is often a sex difference in the degree of stunting associated with undernutrition; in SSA boys are generally affected more than girls (15) an effect which is amplified in lower socioeconomic circumstances. Ethiopia is a large country which has experienced repeated famines, and where 76% of the population live in rural areas with poor socioeconomic conditions. Dietary diversity and micronutrient intake in rural Ethiopia are much worse than in urban areas (14) and stunting is present in 36.6% of Ethiopian under-fives (15) with an excess of affected boys (16).

To explore the effects of rural poverty and undernutrition on type 1 diabetes we have studied urban-rural differences in the disease characteristics among a large register of consecutive, newly diagnosed patients presenting with clinical type 1 diabetes over a 20-year period at diabetes clinics associated with Gondar University Hospital, Northwest Ethiopia. This clinic serves both Gondar city and its satellite rural health centers.

## Materials and methods

### Setting

The study was based in Gondar, Ethiopia, 750 km northwest of the capital, Addis Ababa; a city surrounded by a predominantly rural health zone comprising in total approximately 2.8 million people. All patients were Amhara, the second largest ethnic group in Ethiopia; their language is Amharic, a Semitic language, and phenotypically the Amhara have many Caucasoid features. During the period of this report the diabetes clinic at Gondar University Hospital and 10 rural diabetes clinics were managed by the same consultant physician and clinical team. Rural patients were transferred to the Gondar clinic for initiation of insulin therapy and returned to their rural clinic for long-term follow-up. The same treatment goals were set for all clinics and included continuous patient education. Epidemiological studies of diabetes care and outcomes in this region have been described (1, 4).

### Record study

This case-series included all new patients (n=1682) who presented at the diabetes clinics of Gondar zone over a 20-year period (1996–2016) with rapid onset of classical diabetes symptoms (clinical type 1 diabetes). Ketoacidosis was present in approximately 50% and all were treated with insulin from the first clinic visit. Clinic records included data on residence, age, sex and occupation, together with weight and height (available for 1455 patients). Patients resident outside the Gondar zone were excluded. A sequential sample of 236 newly diagnosed cases of clinical type 1 diabetes (aged ≤35 years) from the main cohort and a similar number of controls (also Amhara) had their demographic and socioeconomic circumstances assessed together with measurements of height, weight and body composition. Analyses of non-fasting C-peptide, diabetes associated autoantibodies, and genotyping for a genome-wide association study (showing associations with type 1 but not type 2 diabetes loci) and HLA-DRB1 alleles have been published from this cohort (4). **Table 1** shows unpublished data from (4) illustrating the differences in socioeconomic conditions between rural and urban cases as assessed in this cohort of 236 cases (data not available on two cases). Rural cases were markedly disadvantaged in education with 31% being illiterate and only 23% completing secondary school compared with 76% of urban cases. Many rural cases were subsistence farmers or laborers, and their housing conditions were very poor compared to the urban cases particularly with respect to access to clean water and fecal disposal. There were no urban-rural differences in the prevalence of antibodies. However, median C-peptide concentrations were markedly lower in the rural than the urban group (0.75 vs. 1.01 µg/l, p<0.001), especially in males (0.72 vs. 1.31µg/l, p=0.008).

**Table 1:** Clinical type I diabetes: Differences between the social and educational background of rural and urban cases, n=234.

Total no. of cases	Rural	Urban	P-value
	205	29	
Educational level:			
Illiterate (%)	63 (31)	0 (0)	<0.001
Secondary or more (%)	48 (23)	22 (76)	<0.001
Occupational group:			
Farmer/labourer (%)	109 (53)	2 (7)	<0.001
Government/business (%)	21 (10)	20 (69)	<0.001
Thatched roof (%)	52 (25)	0 (0)	<0.001
Animals share living space (%)	59 (29)	0 (0)	<0.001
No access to toilet (%)	76 (37)	0 (0)	<0.001
No access to clean water (%)	88 (43)	2 (7)	<0.001

### Statistical analysis

Population data were derived from national censuses carried out in1994 and 2007, and interpolated population estimates used to calculate incidence rates together with 95%CIs based on the Poisson distribution. BMI was calculated as weight (kg) divided by height squared (m^2^) and age and sex-adjusted height and BMI z-scores derived using WHO Anthro software (17). Standard errors for the sex ratio were determined using the binomial distribution. Differences in height and BMI were evaluated using T-tests while differences between discreet variables were analyzed by chi-square or exact tests. Multivariate analyses were carried out with linear regression.

## Results

Over the 20 years a total of 1682 insulin-dependent patients (aged 3 to 82, mean 31.2, SD13.4 yr.) presented in the combined urban and rural areas of Gondar zone. Just 71(4.2%) of these were aged <16 yr. at presentation. The overall annual incidence was 2.75 per 100,000 (95%CI 2.62-2.88). Of these 1100 were males [incidence 3.56 (3.35-3.77) per 100,000] and 582 females [incidence 1.92 (1.77-2.08) per 100,000].

The mean BMI of the adults (18 yr. +) was 18.4 (SD 3.1) kg/m^2^ (M 18.3, F 18.6); 445 (34.0%) were extremely underweight (BMI <17 kg/m^2^), 38 (2.9%) were overweight (BMI 25 to 30 kg/m^2^) and 5 (0.4%) were obese (BMI 30 kg/m^2^ or more).

**Figure 1:**
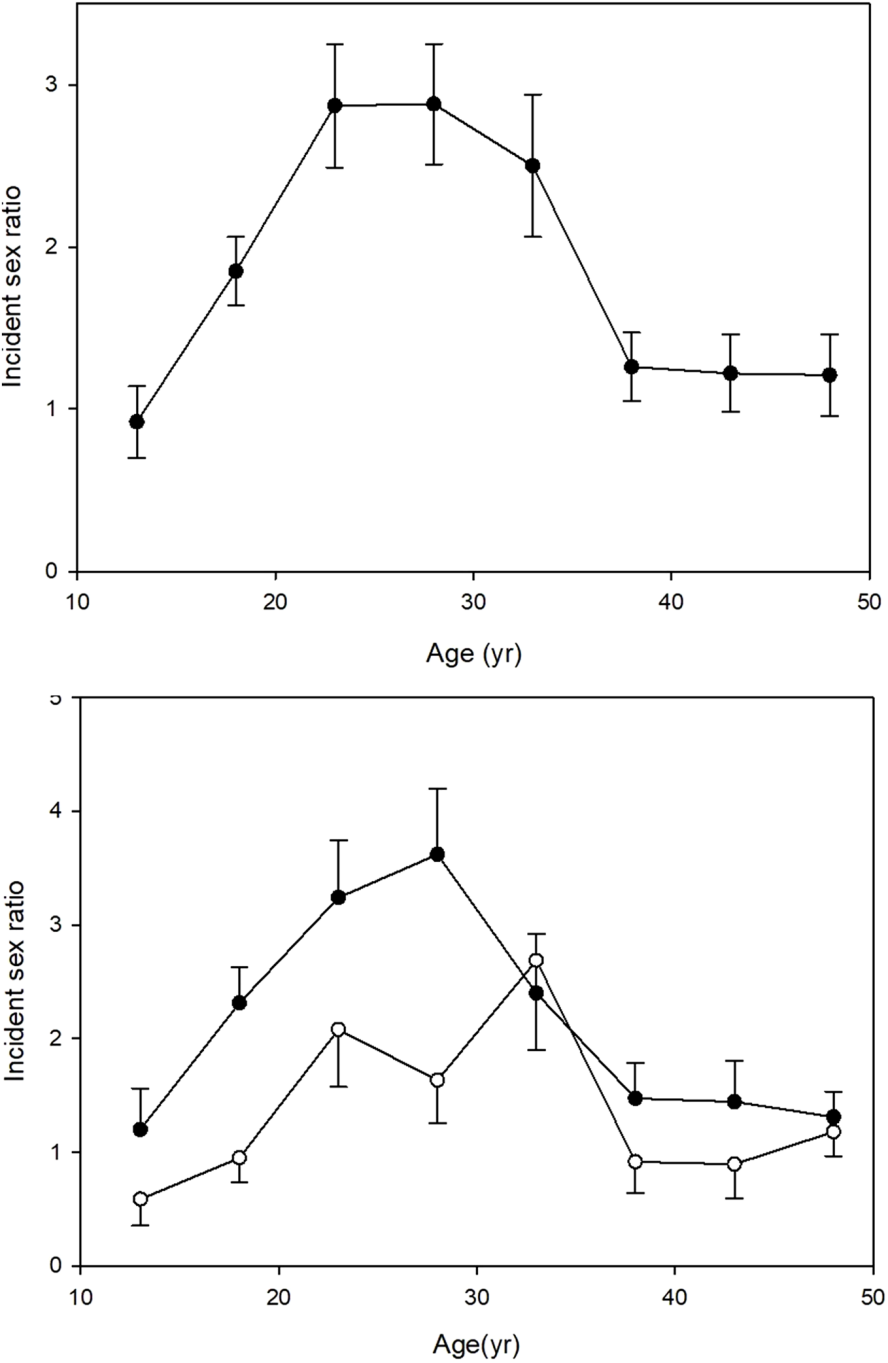
Clinical type 1 diabetes: Male: female sex ratio ( ± SEM) of 1682 cases of newly diagnosed type 1 diabetes cases in Gondar zone over a 20-year period, according to age of onset. Upper panel: all cases. Lower panel: rural cases, closed circles; urban cases, open circles.

**Table 2** shows the differences between the urban and rural cases according to sex. The male excess was much more striking in the rural compared with the urban patients. Of the 1160 rural patients, 804 (69.3%) were male while among the 522 urban patients 296 (56.7%) were male and as a result the rural sex ratio, 2.3M:1F, was significantly greater than the urban sex ratio, 1.3M:1F (P<0.001). The sex ratio was strongly related to age of onset (**Figure 1**, upper panel). It was approximately equal in the <16 yr. age group, rose progressively to a peak at age 20 to 30 yr. and then declined rapidly to reach near equality after the age of 35. The male excess was much greater in rural than the urban patients especially during young adulthood (**Figure 1**, lower panel).

**Table 2:** Clinical type 1 diabetes: Numbers of new cases presenting with rapid onset diabetes-related symptoms and requiring insulin treatment from the outset over a 20-year period in Gondar region according to place of residence (rural or urban) and by gender together with height standard deviation score (SDS), percentage with stunting (height <2 SD), and body mass index (BMI) SDS. (SEM: standard error of the mean).

	Male			Female		p-value
	Rural	Urban	P-value	Rural	Urban	
Total no of cases	804	296		356	226	
Age (SEM)	29.1 (0.4)	33.9 (0.8)	<0.001	31.6 (0.7)	34.6 (1.1)	0.016
No. with Ht/Wt recorded	728	239		322	166	
Height SDS (SEM)	-1.29 (0.04)	-1.00 (0.06)	<0.001	-0.95 (0.05)	-0.95 (0.07)	0.98
No. (%) stunted	151 (20.7)	34 (14.2)	0.026	48 (14.9)	29 (17.5)	0.46
BMI SDS (SEM)	-2.05 (0.06)	-1.53 (0.10)	<0.001	-1.58 (0.08)	-0.88 (0.11)	<0.001

**Table 2** also compares the height and weight measurements within each sex according to whether the patients reported rural or urban residence. The rural men were markedly more growth restricted (lower height z- scores) than urban men (p<0.001) and had a higher prevalence of stunting as indicated by a height SDS score of <-2.0 SD (p=0.026); they also had lower BMI SDS scores (p<0.001). In contrast, the rural women had very similar heights to urban women although their BMI SDS scores were lower than the urban women (p<0.001). In both sexes the rural patients were younger than the urban ones. In regression analyses with height SDS as the dependent variable and rural or urban residence as an independent variable, the effect of rural residence on height was statistically significant (p<0.001) in males but not in females. This was unaffected by the addition of age to the analysis. In further regression analyses again with height SDS as the dependent variable the interaction term (sex*rural residence) was statistically significant (p< 0.02) and remained so after allowing for age in the regression.

**Figure 2:**
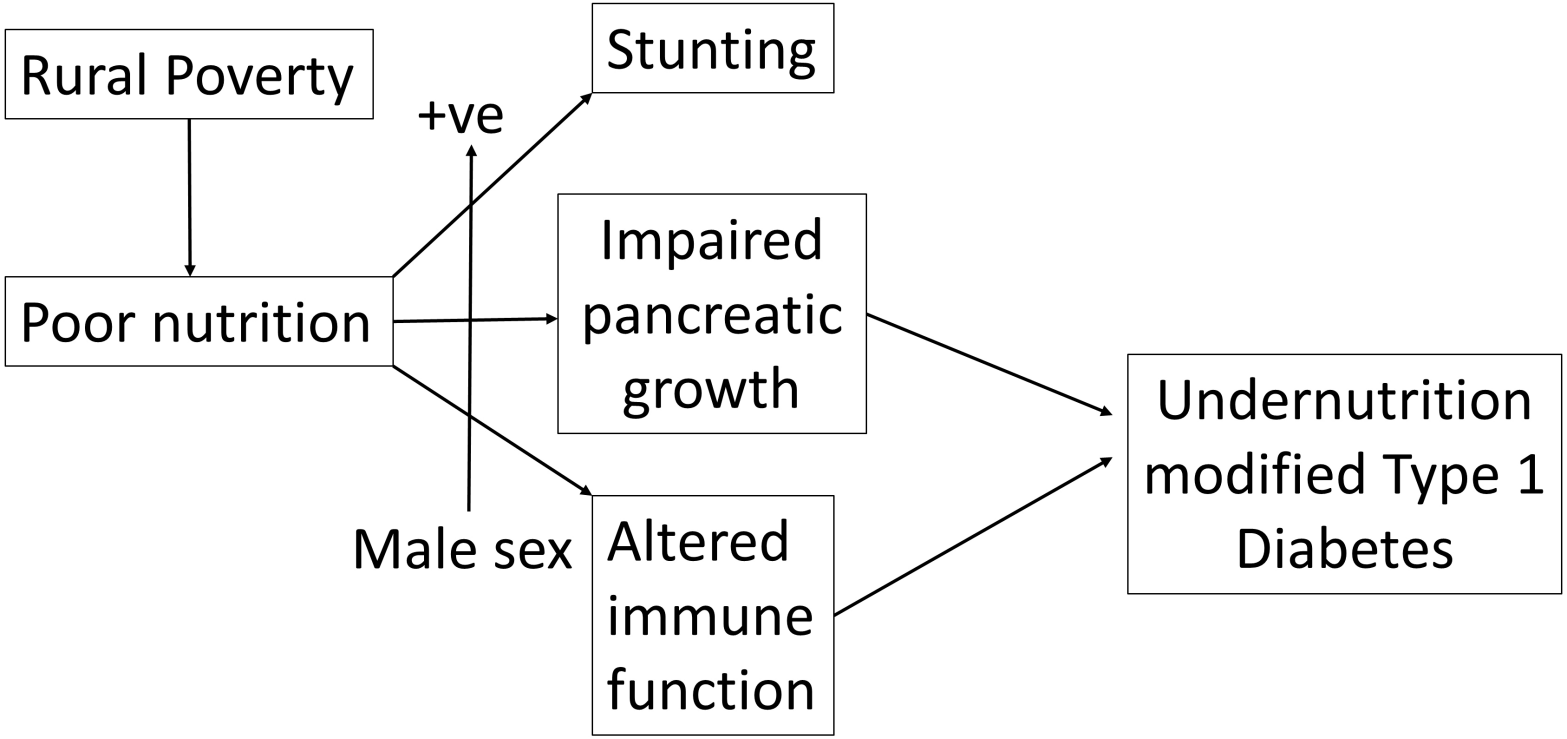
Rural poverty and associated chronic undernutrition interacting positively with male sex leads to both stunting and an altered clinical presentation of insulin-dependent (Type 1) diabetes.

These data have led to the formulation of a hypothesis which is summarized in **Figure 2**. We suggest that rural poverty has a greater effect on males than females resulting in stunting, impaired pancreatic growth and altered immune function which together modify the phenotype of Type 1 diabetes.

## Discussion

This descriptive study of a large group of patients with type 1 diabetes in a predominantly rural area of Ethiopia provides important further evidence for an altered phenotype in this resource-poor community. The annual incidence rates are low but comparable with our previously published estimates (1) and, in part, may be a consequence of under-reporting of the disease particularly in the rural areas because of the difficulties in recognizing and diagnosing the disease where blood glucose measurements are not always available. The relative scarcity of type 1 patients with childhood onset is notable but also consistent with previous observations by ourselves (1, 18) and others in SSA (2, 3). The patients in this study also have very low BMIs with 1/3 meeting a previously suggested criterion for malnutrition-related diabetes (19). The major finding, however, was a striking male excess of presenting cases which was much greater in the rural than urban areas and which was linked with marked growth retardation as indicated by stunting. We argue that the likely explanation is the well-documented, poor nutrition and very poor dietary diversity in this community (13, 14) coupled with the known, but unexplained differential susceptibility to undernutrition of SSA males compared to females, which in turn is much greater in poor socioeconomic conditions (14, 15).

The clearest evidence for undernutrition in the pathogenesis of diabetes comes from animal studies. Reductions in food supply during pregnancy or early postnatal life leads to decreased glucose tolerance and diabetes in the offspring. In rats, a significant reduction in feeding of pregnant mothers during the last week of pregnancy results in low birth weight and 33-56% reduction in pancreatic β-cell mass (20). If suckled by low-protein-fed dams a permanent reduction of β-cell mass results despite being finally weaned on to a control (20% protein) diet (10). This level of protein nutrition is observed in humans in many LMIC. Undernutrition during pregnancy affects the methylation and reduces the activity of many genes that control hepatic and pancreatic function, including growth and transcription factors important for pancreatic development and β-cell differentiation such as *IGF2* (21), *PDX1* (20) and *Hnf4-α* (22), resulting in a permanently reduced β-cell mass and reduced insulin secretion. Altered *PDX1* and *Hnf4-α* function in humans has been associated with Maturity Onset Diabetes of the Young, a non-obese form of diabetes that can be mistaken for type 1 diabetes (23). Deficiencies in the postnatal diet can also affect *IGF2* imprinting in mice (21) and in humans exposed to famine in early life hypermethylation of the *IGF2* gene has been reported (24). Changes in micronutrient levels alone in the peri-conceptional period can alter the methylation of many genes, even when the caloric intake is maintained and in humans, periconceptional micronutrient supplementation with vitamins, minerals and methyl donors is known to alter the methylation status of several genes, of interest is the widespread sex differences among the loci affected (25).

Women of childbearing age and lactating mothers in Ethiopia have many dietary deficiencies, so undernutrition most likely starts *in utero* (26): these deficiencies include vitamin A, vitamin D, vitamin B_12_, folic acid, zinc, iodine, and iron (27–30). Since the conditions related to diet are longstanding in this region of Ethiopia, it seems probable that the fathers of the present cases were also malnourished and may have contributed to changes at conception associated with epigenetic alterations of their sperm (31). Investigation of dietary diversity in the first two years of life has been carried out in detail by others who have shown that there is a major difference between urban and rural areas in SSA, rural areas having lower diversity; furthermore, the urban-rural differences appear to be by far the greatest in Ethiopia compared to the rest of SSA (14). Detailed information on birthweight or postnatal development is not available for the present study as most rural births occur at home. However, local survey data have demonstrated strong links between poor socioeconomic circumstances and low birthweight (32), high prevalence of common childhood illnesses, and poor postnatal growth. Stunting affects 36.6% of the under-five age group with high rates in the rural areas surrounding Gondar (15). The outcomes of early undernutrition depend, at least in part, on both the degree of undernutrition and on subsequent nutritional experience. If early undernutrition is followed by undernutrition during the entire growth period and adult life, with low BMIs in adult life in the absence of acute infectious or diarrheal diseases, the outcomes may be different to a situation where undernutrition is confined to early life. Chronic undernutrition with stunting is most likely to occur during prolonged periods of food insecurity, with repeated famines, and poor food diversity; 76% of the Ethiopian population live in rural, often remote, areas where both macronutrient intake and food diversity is poor, the latter being regarded as very poor (see above). In contrast to rodents, postnatal replication of β-cells in humans is limited. Increase in the human β-cell mass is believed to be very low or absent after five years of age under normal circumstances (33), with minimal increase in response to increased demand as occurs with early obesity (34). However, if promoters of islet cell growth and function such as *PDX1*, *IGF2*, and *Hnf4a* (20–22) are liable to epigenetic inactivation due to undernutrition in antenatal and early life, there will be very little or no increase in β-cell mass in postnatal life. [In human islets from type 2 diabetes patients, increased methylation in relation to *PDX1* resulted in reduced PDX1 expression (35)] Although reduced pancreatic β-cell mass may be sufficient for glycemic control in early life, the deficiency may only become evident during the extra demands of the mid-teen growth spurt, with a greater stress on males who have been most affected by growth restriction. At that point there may be deterioration into insulin insufficiency in the continued presence of undernutrition with rural men worst affected as indicated by the finding of very low C-peptide levels. While this may explain the male excess, it is not clear why young adults are preferentially affected (**Figure 1**) and other factors may be involved. One possibility is that the young adults in our study were exposed to the great Ethiopian famine of 1983-5 during their younger years which affected the majority of the population to a greater or lesser extent (36).

Immune function is also affected by poor diet and poor socioeconomic circumstances and this may alter the presentation of type 1 diabetes. We have previously reported on a small group (n=236) presenting clinically as type 1 diabetes at the Gondar regional clinics (4). In those less than 16 years at onset 86% percent were autoantibody positive, a similar percent to a mainly European group, recently reported (37), it is important to note that both groups were tested shortly after clinical diagnosis. However, autoantibody positivity in the Ethiopians fell off more quickly with increasing age of onset than did the Europeans. Additionally, most of the Ethiopians had single autoantibodies (usually anti-GAD) compared to the mainly European group with multiple autoantibodies. The Ethiopians, all belonging to the Amhara ethnic group, were DR3 and DR4 positive (4). The question arises about what could influence these different autoantibody responses? First, it seems unlikely that it is the result of the well-known genetic diversity in Africa, since the genome of the Amhara people shows least divergence from other non-African groups, including Europeans (38). Second, it is possible that chronic undernutrition which causes immune dysfunction may alter the autoimmune response in type 1 diabetes in the Ethiopians. The immune dysfunction of malnutrition shows in a variety of ways under different circumstances. The effects of malnutrition in children may be seen in the short term with deficient responses (T- and B- lymphocytes) to acute infections (39). Malnutrition may also have long-lasting effects: thus children born in Gambia during the period of greatest food insecurity (‘hungry season’) had smaller thymuses and a higher rate of infection-related deaths in their mid-twenties than those born when food diversity was best (‘harvest season’), suggesting that undernutrition *in utero* has a long-term effect on the immune system (40). Third, in addition to nutrition-associated immune dysfunction, socioeconomic circumstances may contribute: some endemic infections with organisms such as helminths have a restraining influence on the development of autoimmunity. The immune changes initiated by helminth infection are capable of dampening and/or delaying the usual autoimmune response seen in classical type 1 diabetes (41). In rural areas of LMIC including Ethiopia, the absence of protected water sources and toilets favor soil-transmission of helminth infections; the infection rate in NW Ethiopia is high, around 30% (42). In combination with undernutrition-induced immune dysfunction, the delaying effects of helminth infection on the autoimmune reaction may be partially responsible for the low rate of childhood onset type 1 diabetes in rural Ethiopia, in addition to the possibility of some missed diagnoses in remote areas.

## Conclusion

In summary we show that patients presenting with clinical type 1 diabetes in a predominantly rural area of Ethiopia are more likely to be male and that the male excess is greater among rural that urban cases where it is linked with marked growth stunting. Given the prior evidence that undernutrition alters both pancreatic development and immune function (as summarized in **Figure 2**), we suggest that extremely high levels of rural deprivation affect the phenotype of autoimmune type 1 diabetes through complex interactions involving nutrition, growth and the immune system which are much more marked in the male sex.

## Data availability statement

Results on individual patients (anonymized) were derived from clinic records. Requests to access these datasets should be directed to David Phillips, diwp@mrc.soton.ac.uk.

## Ethics statement

No ethics approval was needed for the extraction of anonymised routine data (height, weight, sex, urban-rural) from the outpatient records (n=1682). However, the paper also includes data from a subgroup (n=236) which had previously been investigated in depth. Full ethics approval for this subgroup was obtained from the Gondar College of Medicine and Health Sciences and the UK National Research Ethics Services Committee (REC), reference 14/WA/0132; patients or parents gave their written, informed consent to these investigations. Part of the results from the n=236 study have been published (ref 4), but the socioeconomic data included in this paper have not been published previously.

## Author contributions

SB: Writing – review & editing, Investigation, Resources. DP: Writing – review & editing, Data curation, Formal analysis, Funding acquisition, Writing – original draft. ET: Funding acquisition, Writing – original draft, Writing – review & editing, Conceptualization.
